# Experimental Assays to Assess the Efficacy of Vinegar and Other Topical First-Aid Approaches on Cubozoan (*Alatina alata*) Tentacle Firing and Venom Toxicity

**DOI:** 10.3390/toxins8010019

**Published:** 2016-01-11

**Authors:** Angel A. Yanagihara, Christie Wilcox, Rebecca King, Kikiana Hurwitz, Ann M. Castelfranco

**Affiliations:** 1 Department of Tropical Medicine, Medical Microbiology and Pharmacology, John A. Burns School of Medicine, University of Hawaii at Manoa, Honolulu, HI 96822, USA; wilcoxcl@hawaii.edu; 2 Békésy Laboratory of Neurobiology, Pacific Biosciences Research Center, School of Ocean and Earth Science and Technology, University of Hawaii at Manoa, Honolulu HI 96822, USA; rak62123@hawaii.edu (R.K.); kikiana@go.byuh.edu (K.H.); castelf@pbrc.hawaii.edu (A.M.C.); 3 Department of Natural Sciences, Brigham Young University-Hawaii, Honolulu, HI 96717, USA

**Keywords:** jellyfish, venom, sting, first aid, hemolysis, cubozoan, *Alatina alata*, vinegar, Sting No More™, copper gluconate

## Abstract

Despite the medical urgency presented by cubozoan envenomations, ineffective and contradictory first-aid management recommendations persist. A critical barrier to progress has been the lack of readily available and reproducible envenomation assays that (1) recapitulate live-tentacle stings; (2) allow quantitation and imaging of cnidae discharge; (3) allow primary quantitation of venom toxicity; and (4) employ rigorous controls. We report the implementation of an integrated array of three experimental approaches designed to meet the above-stated criteria. Mechanistically overlapping, yet distinct, the three approaches comprised (1) direct application of test solutions on live tentacles (termed tentacle solution assay, or TSA) with single image- and video-microscopy; (2) spontaneous stinging assay using freshly excised tentacles overlaid on substrate of live human red blood cells suspended in agarose (tentacle blood agarose assays, or TBAA); and (3) a “skin” covered adaptation of TBAA (tentacle skin blood agarose assay, or TSBAA). We report the use and results of these assays to evaluate the efficacy of topical first-aid approaches to inhibit tentacle firing and venom activity. TSA results included the potent stimulation of massive cnidae discharge by alcohols but only moderate induction by urine, freshwater, and “cola” (carbonated soft drink). Although vinegar, the 40-year field standard of first aid for the removal of adherent tentacles, completely inhibited cnidae firing in TSA and TSBAA *ex vivo* models, the most striking inhibition of both tentacle firing and subsequent venom-induced hemolysis was observed using newly-developed proprietary formulations (Sting No More™) containing copper gluconate, magnesium sulfate, and urea.

## 1. Introduction

Jellyfish envenomations, which have been increasing worldwide, result in myriad clinical outcomes, ranging from sting-site pain and inflammation to life-threatening sequelae and death [1]. In response to heightened public health concerns and lay inquiries, clinicians and emergency-care personnel often rely on tertiary Internet resources, such as Medscape, eMedicine Health, or Mayo Clinic Online [2,3,4]. These clinical management summaries cite an assortment of primary peer-reviewed references, many of which are contradictory and/or uncorroborated [5,6,7]. Further confusion results from authoritative Internet articles advising both clinicians and the general public [8], which often echo and extrapolate from selected studies without critically discussing pertinent limitations or divergent results. For example, recent articles have uncritically perpetuated already-hyperbolic press releases based on small-scale, *in vitro* model studies [9] into headlines declaring how vinegar “can be deadly” and “may kill” [10,11], suggested disproven treatments like ice packs [8,12], and recommended the use of shaving cream [8] and/or sting-site scraping, which have never been corroborated by any human study or validated model [13,14] and involve the application of site pressure which has been shown to worsen outcomes. The confused lay press recommendations and lack of validated protocols are especially dangerous in the case of certain cubozoan envenomations, which cause more loss of life than shark attacks annually [15,16,17,18].

Rigorous, non-redundant and mechanistically distinct testing of the efficacy, as well as the potential harmful side effects, of many jellyfish treatment options is important because animal models indicate that the pathology is venom dose-dependent and, thus, if treatment methods increase venom dose or “load”, they may cause more harm than good [19,20,21]. Additionally, since emergency responders may not have standardized approaches available, especially in remote areas, it is important to know what other options cause the least harm.

However, because jellyfish envenomations, and particularly cubozoan envenomations, are potentially dangerous, live tentacle application involving human participants to comparatively assess different first-aid measures is inherently hazardous. While the potency of the tiny carybdeid, *Carukia barnesi*, to cause Irukandji syndrome, used investigator self-testing and self-consenting tests [22], broader human studies are ethically problematic. Mouse and piglet animal models have been extremely valuable in the elucidation of pathogenic mechanisms [19], but low cost and reproducible *ex vivo* and *in vitro* laboratory-based models are critically needed to accelerate progress and rigorously investigate approaches to mitigate cnidae discharge and venom activity in the determination of best practices. Ideal models would be broadly applicable, reproducible, easy-to-measure, and accurately recapitulate sting events. To defensibly correlate to the biological reality of an authentic sting, ideal models would meet the following criteria:

(1) Exhibit spontaneous tentacle cnidae discharge. We observed that application of live, freshly-cut tentacle sections onto low melting point agarose gel sections made up with fresh, intact human red blood cells (RBC) elicited an immediate potent sting response with discharged nematocyst tubules visibly penetrating deep into the blood agarose, as well as tentacle contraction, indistinguishable from spontaneous stinging after application of tentacle sections on human skin [23]. Artificial stimuli can induce explosive sting-type events in which essentially all venom-filled penetrant cnidae (nematocysts) discharge or non-productively rupture. Specifically, electrical (DC voltage) stimulation causes massive circumferential tentacle nematocyst discharge, as well as visible tentacle damage with heat effects, and is in extreme excess of physiological electrical excitation [24,25]. Chemical stimuli, such as exposure to alcohols, can also cause nearly all tentacle nematocysts to explosively fire venom-delivering hypodermic-like nematocyst tubules [26]. But in both cases, nearly all cnidae are fired and other tentacular cell types are disrupted or lysed. Thus, the toxic exudate from either approach differs markedly from authentic sting-associated venom both quantitatively and qualitatively.

(2) Allow for visualization and quantification of cnidae discharge. Envenomations involve the firing of up to thousands of packed cnidae per linear tentacle unit of length. It is critical that potential first-aid approaches to remove adherent tentacles be tested to examine whether a specific approach reduces or induces additional cnidae discharge. An optimal envenomation model must allow for imaging and ideally quantification of cnidae discharge with which to experimentally assess test conditions (e.g., topical temperature or pressure) or test solution efficacy to prevent, inhibit, or exacerbate tentacle cnidae discharge.

(3) Measure venom activity directly. An ideal model of envenomation would allow direct and time-dependent determination of venom activity in the experimental tissue model. The hemolytic activities of jellyfish venoms have been well-studied, and hemolytic units (specific activity per venom mass) for different species have been calculated [20,21]. Such biochemical baselines provide clear standards for envenomation models. Experimental models that do not result in levels of venom activity comparable to primary or laboratory purified venom samples are not credible proxies for sting events.

(4) Employ rigorous controls. It is also critical to investigate the effects of comparative controls including null treatment or the application of a control “mock” solution such as seawater to exclude experimental artifacts, including direct physical manipulation effects of adding solutions in treatment activities. Such controls are especially important given that there is no careful study to determine what percentage of cnidae discharge during natural sting events; far less than 100% of the cnidae present on a tentacle may actually fire. It is also critical to assess converse interpretations of results, such as whether a tested solution may enhance venom recovery from tissue and thus reduces “venom load”, or alternatively, whether site pressure alone from the application of a treatment causes or enhances venom expulsion from already-discharged nematocysts [27]. In the later example, the amount of venom injected simply by the application of the first-aid measures or sting-site “scraping” should be compared to the venom injected and potentially injected without such measures in efforts to ascertain treatment choice and effectiveness.

Previous studies have sought to test first-aid measures in a diversity of cnidarian species [28,29,30,31,32,33,34,35,36,37,38,39,40,41,42,43,44,45,46,47]. However, these experiments varied widely in methods and results, and none employed comprehensive approaches, which met the four above-mentioned criteria simultaneously. In this study, we have employed an array of experiments to rigorously test first-aid approaches from multiple angles. In addition, we unveil a novel *ex vivo* model, which alone is able to meet the four requirements listed above. Apart from satisfying these criteria, the model is simple and inexpensive and, thus, could be employed in laboratories with minimal budgets and limited equipment.

## 2. Results and Discussion

### 2.1. Model Validation

#### 2.1.1. Live Tentacles Spontaneously Sting and Imaging

**Figure 1:**
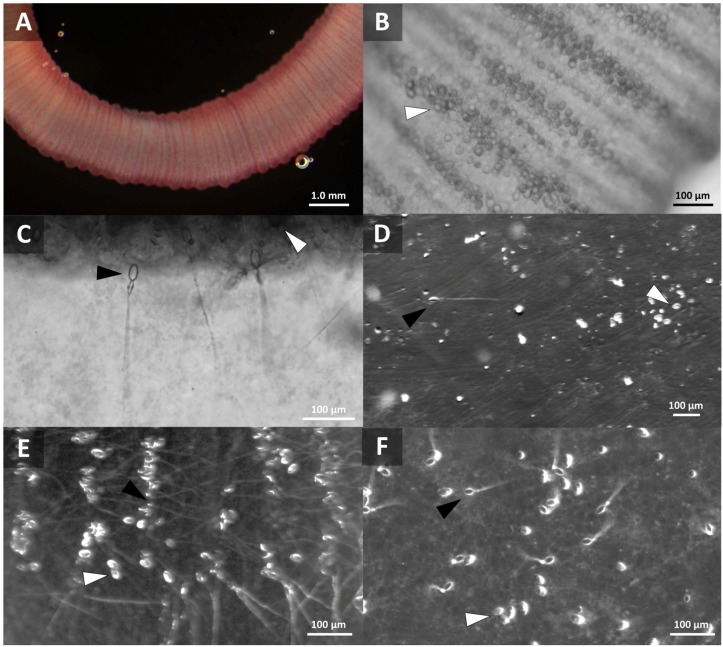
Visualization of spontaneous stinging in *ex vivo* models. Black arrows indicate examples of discharged cnidae with impaling tubules visibly penetrating agarose; white arrows indicate non-discharged cnidae. (**A**) Live *A. alata* tentacle with visible cnidae bands; (**B**) Higher magnification to show cnidae bands and allow quantification of tentacle cnidae; (**C**) Cnidae discharge from a live tentacle into blood agarose (TBAA); (**D**) Cnidae remaining on blood agarose after tentacle removal (TBAA); (**E**) Cnidae remaining on artificial “skin” after tentacle removal (TSBAA); (**F**) Cnidae lifted from human skin after a sting using the sticky tape method.

Our models (described in detail in “3. Experimental Section”) employed tentacles cut from freshly-caught, live animals to most accurately recapitulate sting events (Figure 1A). We were able to visualize undischarged cnidae in the tentacle prior to stinging (Figure 1B), as well as directly observe cnidae discharge from tentacles into the blood agarose substrate (Figure 1C). We also were able to determine cnidae discharge after tentacle removal; tentacles were found to spontaneously sting, on contact, both blood agarose (Figure 1D) as well as cleaned porcine small intestine (which was used as a “skin” proxy) laid over blood agarose (Figure 1E). The cnidae visible after tentacle removal were not qualitatively different from cnidae detectable on human skin after actual stings, removed using the sticky-tape method (Figure 1F) [48].

#### 2.1.2. Quantification of Cnidae Discharge

Quantification of cnidae discharge in a human sting event was performed by placing 1-cm lengths of *A. alata* tentacle onto human skin and examining the tentacle itself before and after to estimate percent discharge of cnidae (Figure 1B). The number and percent of discharged cnidae left behind on the skin were determined using the sticky tape method. We found that the vast majority of cnidae do not discharge. After accounting for tentacle contraction, counts of cnidae density on the tentacle before and after 5 min of stinging were not significantly different. The number of cnidae left behind on the skin was several orders of magnitude lower than the total cnidae in the tentacle, and only a portion of those adherent cnidae were discharged (Table 1). These results indicated that a very small number of cnidae are responsible for the pathology associated with stings. For this reason, first-aid priorities should be to inhibit discharge of intact cnidae both in adherent tentacles as well as shed onto the skin.

Results of tentacle cnidae counts and adherent cnidae from our TBAA paralleled those from human stings with no significant difference in the total cnidae left adherent on the agarose or the number that discharged (Table 1).

**Table 1:** Comparison of cnidae adherence and discharge between human stings (*N* = 4) and the TBAA (*N* = 8).

Sting Substrate	Tentacle Cnidae/mm^2^	Cnidae Adherent (Total per mm^2^)	Cnidae Discharged (Total per mm^2^)	Percent of Tentacle Cnidae Discharged
Human Skin	1055–1355	2.6–25.3	0.4–4.5	0.03%–0.43%
	*1200 ± 68*	*9.3 ± 1.8*	*1.5 ± 1.0*	*0.21% ± 0.10%*
TBAA	804–1499	4.5–42.2	1.0–6.3	0.07%–0.46%
	*1211 ± 97*	*14.0 ± 4.2*	*3.3 ± 0.7*	*0.24% ± 0.05%*

Range is listed with average ± standard error below in italics. No significant differences could be found between the data sets using two-tailed, heteroscedastic *t*-tests.

#### 2.1.3. Quantification of Envenomation Triggered Hemolytic Activity in Blood Agarose

Previously developed biochemical methods of venom recovery from isolated cnidae [19] result in reproducible specific activities of approximately 20 ng/HU_50_ unit (HU_50_ is the mass required to lyse 50% of RBC of a 1% RBC solution in 1 h at 37 °C). Based upon total cnidae recovered and final yields, this biochemical purification method typically yields approximately 0.06 HU_50_ per cnidae.

**Figure 2:**
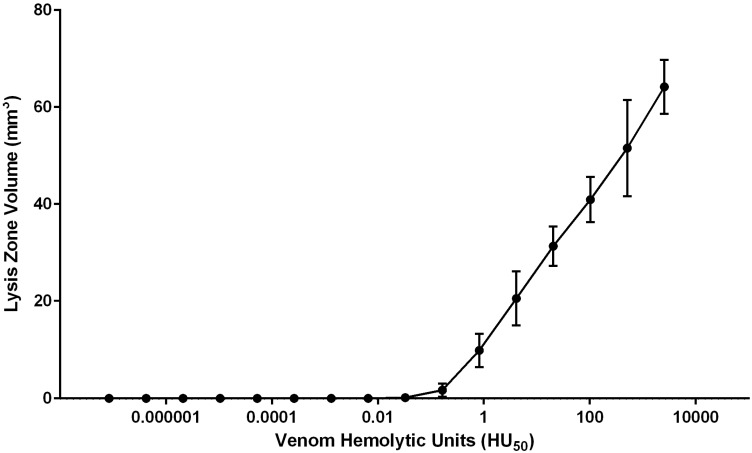
Semi**-**logarithmic correlations between venom hemolytic units and hemolytic zone size (mm^3^). The hemolytic zone was dose-dependent in a logarithmic manner.

We sought to quantitate the hemolytic activity released *in situ* from live and intact tentacle envenoming cnidae using a blood agarose spontaneous envenomation model (TBAA) in which hemolysis could be clearly observed by the disruption of light scattering intact red blood cells and the formation of clear zones of hemolysis. The correlation of hemolytic units (HU_50_) to lytic volume in the blood agarose model was examined. Specifically, biochemically-purified venom [19] was applied to wells in blood agarose to determine the reproducibility of dose-dependent area of hemolysis (VBAA, Figure 2).

**Figure 3:**
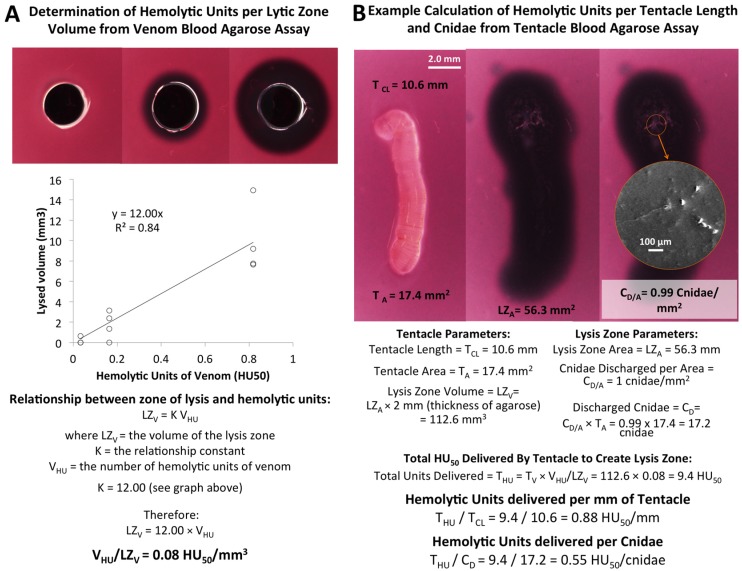
Mathematical deduction of hemolytic units per cnidae and hemolytic units per millimeter of tentacle. Well diameter is 2 mm. (**A**) Derivation of hemolytic units required to lyse 1 mm^3^ of 1.0% RBC in 1.5% agarose after one hour at 37 °C from the linear portion of the dose-response curve; (**B**) Derivation of hemolytic units delivered per millimeter tentacle and per cnidae, based on tentacle measurements, counts of discharged cnidae, and the hemolytic units per volume calculated in A.

The number of hemolytic units (HU_50_) required to lyse 100% of a 1 mm^3^ volume of 1% RBC in 1.5% agarose was determined from the linear portion of the curve (using *R^2^* values). The equation for the linear dose response was *y* = 12.00*x*, where *y* is the volume of blood agarose lysed (LZ_V_) and *x* is the HU_50_ dose, resulting in an estimation of 0.08 HU_50_ required to lyse 1 mm^3^ (Figure 3A).

To determine the minimum number of units delivered per millimeter of stinging tentacle, the relaxed tentacle length, width, and cnidae density were determined prior to tentacles being laid on blood agarose (Table 1). After tentacles spontaneously stung for five minutes, contracted length (T_CL_), width, and area coverage (T_A_) were recorded. Once tentacles were removed, their final contracted length, width, as well as cnidae density, were again determined. The density of discharged cnidae under the tentacle per unit area (C_D/A_, Figure 3B) was determined from direct microscope image counts. Using T_A_ multiplied by the C_D/A_, we calculated the total number discharged cnidae (C_D_) for each tentacle. After one hour at 37 °C, the area (LZ_A_) and volume (LZ_V_, Figure 3B) of 100% lysis was determined as well as the hemolytic units necessary for the volume lysed (Figure 3A). The average hemolytic units (based upon *N* = 8 tentacle sections) was 0.3 ± 0.2 HU_50_ (Table 2) per cnidae and 1.0 ± 0.2 HU_50_ per mm tentacle.

**Table 2:** Range and mean values for tentacle length, volume of lysis at one hour, discharged cnidae per unit area, hemolytic units per unit tentacle length, and hemolytic units per cnidae (*N* = 8).

Tentacle Length (T_CL_)	Volume of Lysis (LZ_V_)	Cnidae Discharged per mm^2^ (C_D/A_)	Hemolytic Units per mm Tentacle	Hemolytic Units per Cnidae
6.45–10.61 mm	65.7–118.6 mm^3^	1.0–6.3 cnidae/mm^2^	0.7–1.4 HU_50_/mm	0.1–0.5 HU_50_/cnidae
*8.55 ± 0.61 mm*	*99.2 ± 6.2 mm^3^*	*3.3 ± 0.7 cnidae/mm^2^*	*1.0 ± 0.08 HU_50_/mm*	*0.3 ± 0.06 HU_50_/cnidae*

Range is listed with average ± standard error in italics.

Authentic envenomation by live cnidarians involves the direct injection of full potency venom. An ideal model would recapitulate this specific activity. Due to limitations in cnidae recovery as well as venom lability and autolysis by venom proteases, biochemical technique based methods only allow for fractional recovery of authentic specific activity. In contrast, methods to quantitate the specific activity of spontaneous, live-tentacle discharged venom were examined to more closely estimate specific activity doses comprising authentic envenomations.

From these tentacle-based calculations, potential doses delivered into sting victims were derived. Venom dose was expressed as the number of hemolytic units per mL of blood volume per percent RBC (U/mL/%) [19]. Based on calculations from Table 2, 1 m of *Alatina alata* tentacle could envenom between 700 and 1400 HU_50_. If we extrapolate to a human with a blood volume of 5 L and hematocrit of 38%, then the total dose delivered upon 1 m of tentacle contact would be between 0.0037 and 0.0074 U/mL/%. While these approaches at quantitation represent a significant step towards authentic pathophysiological modeling, it should be noted that the agarose model does not contain any type of circulatory system and venom is fully constrained by limited diffusion through agarose. Furthermore, the low RBC percent content of the agarose model also leads to an underestimation of the full lytic potential of venom delivered per mm tentacle contact. Despite these model-based limitations, there is significant utility in this model based on the potential to examine various approaches to mitigate stings in direct assay of fresh tentacle-based effects on human blood.

#### 2.1.4. Rigorous Controls

The Tentacle Skin Blood Agarose Assay (TSBAA) satisfied the four requirements of a good envenomation model: (1) it utilized the natural stinging activity of live tentacles rather than electrically-forced or chemically-induced discharge and, thus, more accurately reflects the amount of venom discharge during a natural sting event; (2) it allowed for visualization of cnidae discharge through microscopic examination; (3) it used hemolysis, a well-categorized venom activity, to directly evaluate sting outcomes; and (4) it allowed rigorous controls, including withholding of treatment as well as inert treatments, such as seawater or the application of heat or cold.

**Figure 4:**
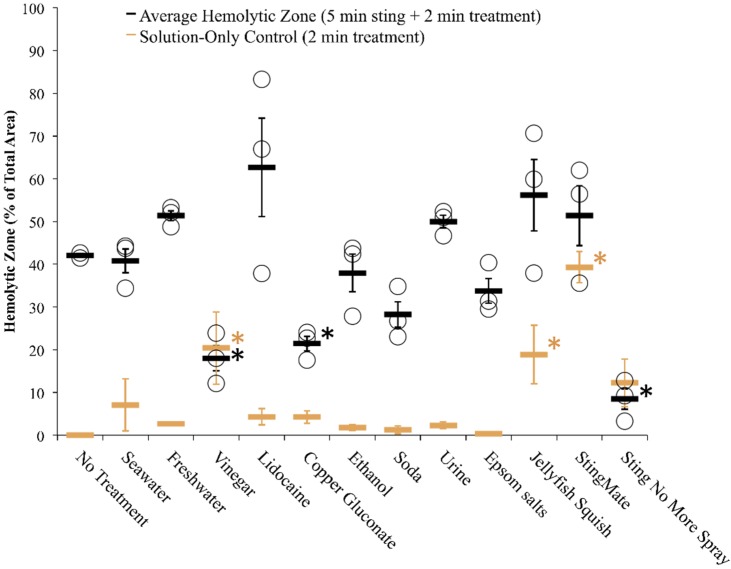
Size of hemolytic zone following post-sting treatments using the TBAA. Significant differences were found among solutions in both control and experimental datasets (one-way ANOVA; *p* < 0.0001 for both; significant differences from no treatment using Holm’s *post hoc* multiple comparison test indicated with color-coded asterisk, *). Significant lysis in controls for vinegar, Jellyfish Squish^®^, and StingMate^®^ (*p* < 0.05 for all, from Holm-Sidak *post hoc* multiple comparison test) revealed limitations of the blood agarose model (solution-induced lysis), prompting the development of a skin proxy. Black asterisks indicate a significant difference between hemolysis due to tentacle exposure alone as compared with tentacle caused hemolysis after treatment with listed test solution.

The simpler Tentacle Blood Agarose Assay (TBAA), initially attempted, satisfied two of the three requirements of a good envenomation model, as it also met the first three requirements. However, the application of rigorous controls revealed a high degree of solution-induced hemolysis or hemoglobin oxidation (due to pH or osmotic-based effects on human RBC) and/or effect-masking color change, which does not occur when these solutions are applied over a barrier such as human skin (Figure 4). Thus, the addition of a “tissue layer” to simulate skin was essential to most accurately recapitulate a sting event.

To create a “skin” for blood agarose, published protocols for pig-derived skin grafts were used (see Methods). Spontaneous tentacle stinging and cnidae discharge were not inhibited by the presence of the “skin”; however, the “skin” provided an effective barrier function such that topical application of vinegar and other solutions no longer caused solution-induced hemolysis.

### 2.2. Evaluation of First Aid Approaches

#### 2.2.1. Tentacle Solution Assay (TSA)

Firstly, we evaluated the potential to inhibit nematocyst discharge (thus reducing venom dose in the victim) using the TSA; and secondly, we directly assessed solution effects on hemolytic activity as a measure of envenomation-related tissue damage and/or pain (TSBA). Ideal therapeutic approaches would be readily available, inexpensive, and perform well using both metrics across multiple species.

**Figure 5:**
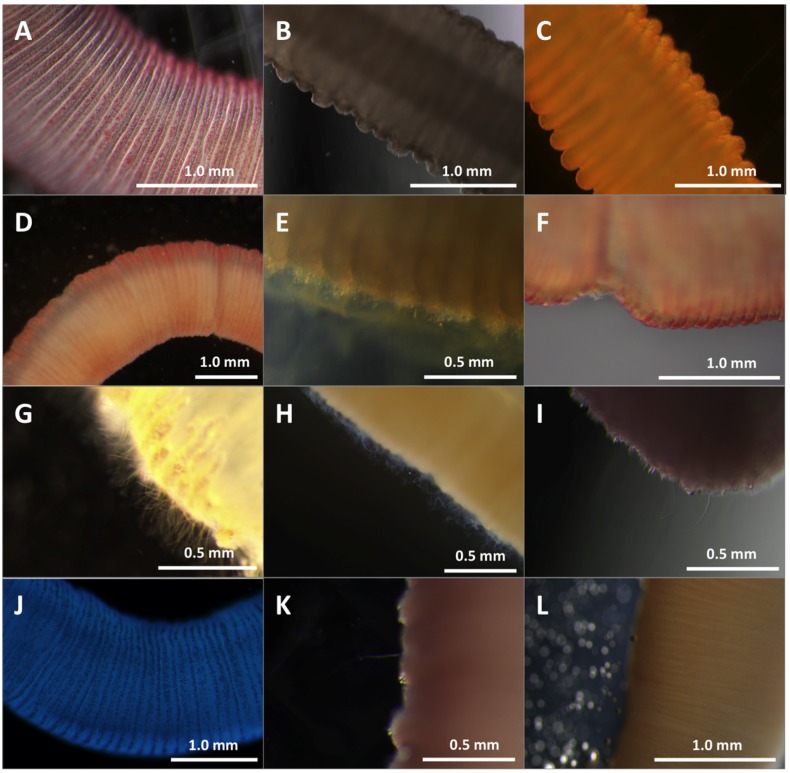
Effects of test solutions in TSA. Cnidae discharge from tentacles after 10 min in different test solutions. (**A**) seawater; (**B**) vinegar; (**C**) lidocaine; (**D**) urine; (**E**) cola carbonated soft drink; (**F**) saturated Epsom salts; (**G**) isopropanol; (**H**) ethanol; (**I**) freshwater; (**J**) Sting No More™ Spray; (**K**) Jellyfish Squish^®^; (**L**) StingMate^®^. Seawater, vinegar, lidocaine, and Epsom salts (**A**–**C**,**F**) did not induce cnidae discharge. Urine (**D**) and freshwater (**I**) induced a small degree of discharge over time. Soda (**E**) caused immediate degradation of the tentacle with cnidae discharge creating a mat on the tentacle surface. The isopropanol and ethanol (**G**,**H**) caused immediate, massive cnidae discharge. None of the commercial products (**J**–**L**) induced cnidae discharge. Scale bars as shown.

Solutions had significantly different effects on cnidae discharge (one-way ANOVA *p* < 0.05; Table 3 and Table 4, and Figure 5). Lidocaine, vinegar, and copper gluconate did not induce nematocyst discharge below seawater controls, while urine and isopropanol led to dramatic, significant increases in nematocyst discharge (*p* < 0.01 for both; Table 4). Freshwater, ethanol, a saturated Epsom salt solution and soda-induced moderate non-significant nematocyst discharge when compared to seawater (Table 4). Vinegar and copper gluconate prevented isopropanol-induced cnidae discharge when tentacles were pretreated for as little as one minute, while vinegar alone also protected against pressure-induced cnidae discharge (Table 3).

**Table 3:** Percent cnidae discharge from test solutions either alone (10 min incubation), or after 1 min incubation followed by discharge stimulants (*N* = 3).

Test Solution	Solution-Induced Discharge	Isopropanol-Stimulated Discharge	Pressure-Stimulated Discharge
Seawater	1.8% ± 1.8%	42.4% ± 9.3% ^§^	47.2% ± 4.7% ^§^
Vinegar	0.3% ± 0.3%	2.1% ± 1.2% ^†^	0.0% ± 0.0% *
Lidocaine	0.2% ± 0.2%	70.5% ± 5.3% ^§^	31.8% ± 6.6% ^§^
Copper Gluconate	0.0% ± 0.0%	13.2% ± 3.7% ^†^	40.4% ± 7.7% ^§^
Isopropanol	52.1% ± 13.2% *	-	82.3% ± 2.0% *

Discharge was induced either chemically by 10 min of incubation with isopropanol, or physically by pressing the tentacle between two coverslips for 10 seconds. Values listed are average ± standard error. Significant differences were detected between solutions and between non-stimulated and stimulated tentacles using a two-way ANOVA (*p* < 0.01), with solution accounting for 46.9% of the variation and stimulation accounting for 16.9% of the variation. * indicates solution discharge significantly different from seawater-induced discharge with the same stimulant (*p* < 0.05 using Holm-Sidak *post hoc* multiple comparison test); ^†^ indicates chemically-induced discharge is significantly different from isopropanol alone; § indicates significant increase in discharge as a result of stimulant when compared to the respective test solution alone, and thus lack of discharge inhibition. Vinegar strongly inhibited both chemically- and pressure-stimulated cnidae discharge, while copper gluconate only prevented chemically-induced discharge.

#### 2.2.2. Tentacle Skin Blood Agarose Assay (TSBAA)

**Figure 6:**
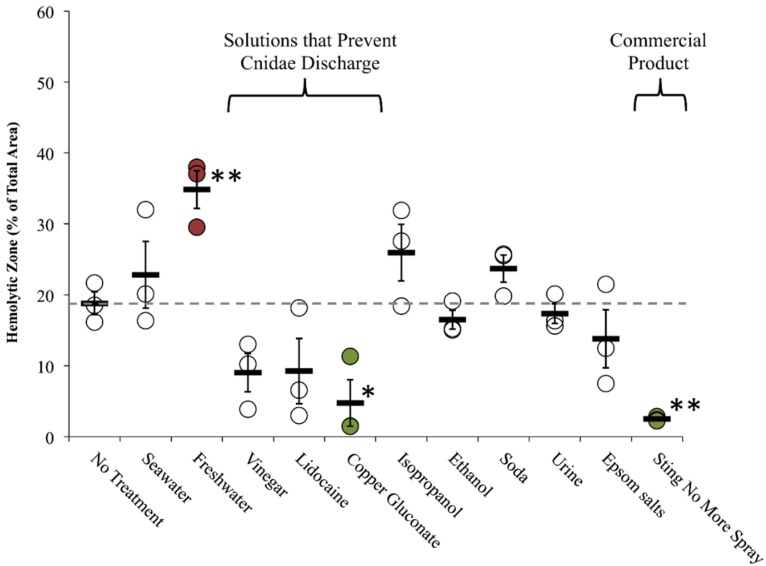
Rinse solution effects (on-tentacle) in TSBAA. Size of hemolytic zone following tentacle removal solution tests: solutions added 3 min into a total sting time of five minutes; grey dashed line indicates average hemolytic area with no treatment. Solutions that prevented cnidae discharged had smaller average percent areas. Significant differences were found between rinse solutions (one-way ANOVA, *p* < 0.01; asterisks indicate significant difference from no treatment using a Holm-Sidak *post hoc* multiple comparison test; * means *p* ≤ 0.05, ** means *p* < 0.01). Commercially available Sting No More™ Spray and one of the active ingredients, copper gluconate, significantly reduced hemolysis (highlighted in green), while freshwater significantly exacerbated hemolysis (highlighted in red).

Envenomation involves both direct trauma to the epidermis and dermis by microscopic, spine-laden, hollow tubules from the explosive discharge of thousands of penetrant cnidae, or nematocysts, per millimeter tentacle contact, as well as the biochemical effects of the injected complex venom. Thus, first-aid approaches may reduce sting severity by (a) preventing undischarged nematocysts from firing and causing further trauma or (b) reducing the activity of venom already injected. First aid solutions which accomplish (a) might be used to remove adherent tentacles or to rinse the area after a sting to inactivate remaining nematocysts. Our evaluation of first aid approaches was designed to investigate test solution effects on both aspects of envenomation. To evaluate potential tentacle removal solutions, we tested whether they would cause an increase or decrease in discharge when added directly to adherent, stinging tentacles. Significant differences between treatments were detected (one-way ANOVA, *p* < 0.01). As expected from the lack of discharge seen in the TSA, vinegar, lidocaine, copper gluconate and Sting No More™ Spray reduced the area of the hemolytic zone, however, only the latter two were significantly different from no treatment (*p* < 0.05 and *p* < 0.01, respectively, using Holm-Sidak *post hoc* test with planned comparisons; Figure 6). Freshwater increased hemolysis (*p* < 0.01), while seawater, urine, soda, ethanol, and isopropanol failed to mitigate hemolysis.

**Figure 7:**
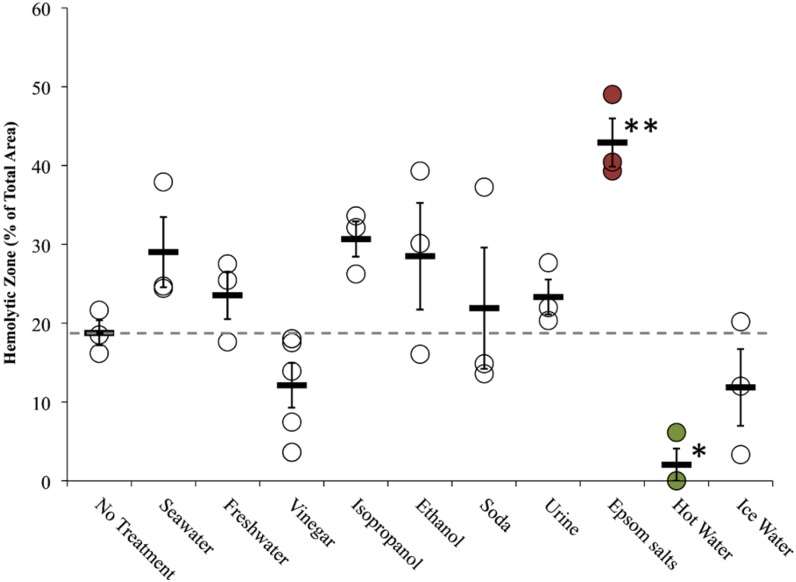
Treatment effects (after tentacle removal) of readily available first aid options in TSBAA; grey dashed line indicates average hemolytic area with no treatment. Significant differences were found between solutions (one-way ANOVA, *p* < 0.01; asterisks indicate significant difference from no treatment using a Holm-Sidak post-hoc multiple comparison test; * means *p* = < 0.05, ** means *p* < 0.01), with Epsom salts significantly exacerbating hemolysis (highlighted in red). Significant differences were also found between post-sting temperature treatments (one-way ANOVA, *p* = 0.03). 5 min with a hot water compress significantly reduced the hemolytic zone (highlighted in green), while an ice water compress did not.

To evaluate post-sting treatments, tentacles were allowed to sting for the full five minutes, removed manually, after which first aid solutions were added to the sting area and allowed to act for two minutes. To test the effects of temperature, hot and cold compresses were added after tentacle removal and left for 5 min. Of the readily available sting treatment options, only hot water significantly reduced hemolysis (Figure 7; one-way ANOVA *p* = 0.03 with *post hoc* Holm-Sidak test *p* < 0.05). While there were significant differences between first-aid solutions and no treatment (one-way ANOVA, *p* < 0.01), this was driven by significantly worse hemolysis from the application of Epsom salts (*p* < 0.01).

**Figure 8:**
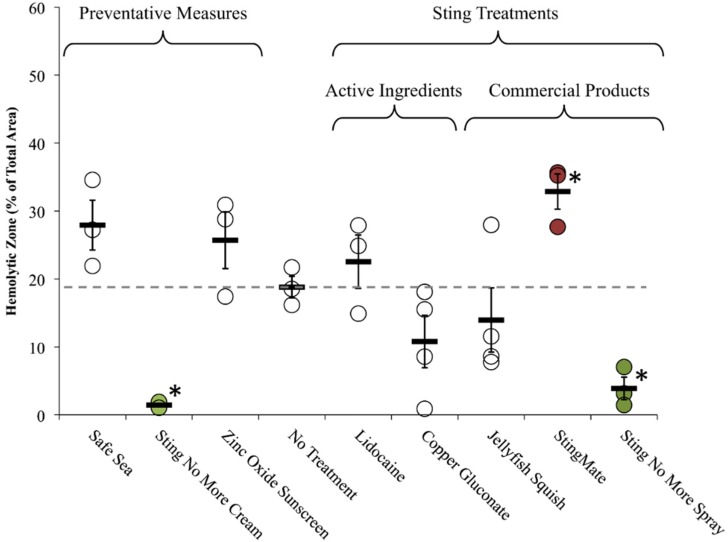
Effects of commercially-available preventive measures and post-sting first aid on hemolytic zone in TSBAA; grey dashed line indicates average hemolytic area with no treatment. Significant differences were found between preventative measures (one-way ANOVA, *p* < 0.01; * indicates significance from no treatment of *p* < 0.05 using Holm-Sidak *post hoc* multiple comparison test). Only Sting No More™ Cream significantly reduced the hemolytic area (highlighted in green). There were also significant differences between commercial products applied sting treatments (one-way ANOVA, *p* < 0.01). Sting No More™ Spray significantly reduced hemolysis (highlighted in green), while StingMate^®^ significantly exacerbated hemolysis (highlighted in red).

The commercially available products differed greatly in their ability to reduce hemolysis over time. StingMate^®^ (First Aid Mates, LLC, Jackson, MS, USA) actually increased hemolysis, while Jellyfish Squish^®^ (Coastal Solutions Inc., Savannah, GA, USA) led to an approximate 25% reduction in hemolysis (not significant), and Sting No More™ (Alatalab Solutions, LLC, Honolulu, HI, USA) Spray treatment reduced hemolysis by more than 80% (Figure 8).

**Figure 9:**
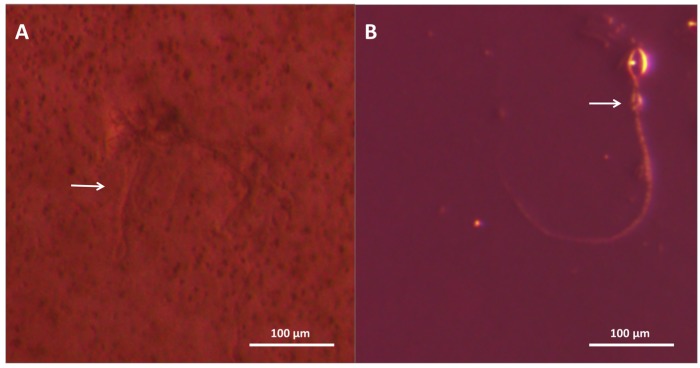
Microscopy images of blood agarose treated with (**A**) Sting No More™ and (**B**) seawater. Arrows point to discharged cnidae; cnidae capsules are approximately 10 × 20 microns. In (**A**), several discharged cnidae sit among RBC, showing that the product did not prevent discharge but did protect against the discharged venom’s hemolytic effects, whereas in (**B**), there are no RBC surrounding the cnidae, suggesting the venom extruded lysed all cells in the vicinity.

Finally, we also tested pre-treatments that claim to prevent stinging from occurring as well as non-chemical post-sting first aid approaches. As a preventative measure, SafeSea^®^ (Nidaria Technologies Ltd., New York, NY, USA) failed to prevent stinging or sting-induced hemolysis, and did not fare better than an ordinary sunscreen. Sting No More^TM^ Cream, on the other hand, inhibited hemolysis. Microscopic examination revealed that while the cream did not fully inhibit cnidae discharge, application of the cream fully prevented RBC lysis despite direct and proximal exposure to the venom hemolytic components (Figure 9).

#### 2.2.3. Informed First-Aid Treatments

Previous studies on first aid approaches to jellyfish envenomations have been narrowly focused, approaching the question from a limited number of angles (Table 4 and Table 5). Such single-lens approaches are prone to error, particularly when they do not meet the standards of an ideal model including natural stinging, quantification of both cnidae discharge and venom load, and rigorous controls. Our models allow for an array-based approach, thus allowing for the evaluation of the solution from a diversity of angles. This array-based approach allowed us to evaluate previous conflicting results and explore why such conflicts arose.

Globally, the standard approach for the removal of adherent tentacles includes the liberal application of vinegar [17,18]. Previous studies have shown that weak acetic acid solutions (including commercial vinegar) irreversibly inhibit nematocyst discharge in cubozoa [28]. However, two studies reported a slight increase in nematocyst discharge in *Chrysaora* tentacles after the application of 5% acetic acid [32,34]. Welfare and colleagues reported that the application of vinegar to adherent tentacles after electrically-stimulated discharge resulted in slight recovery of additional cytolytic activity after a seawater wash and made the extrapolated claim that vinegar could increase venom discharge from already-fired nematocysts, potentially leading to a higher “venom load” in sting victims [47]. However, the low relative toxicity recovered from this post-vinegar wash (~1% of previously reported primary specific activity) calls into question its relevance, and with no control solutions tested, it is impossible to determine if the recovery represents further discharge from vinegar application or simply the pressure of any treatment application.

Our study supported previous findings that vinegar precludes nematocyst discharge (Table 3), and did not corroborate claims that vinegar increased “venom load” or primary activity in our sting models (Figure 5 and Figure 7 and Table 4). In fact, the opposite effect was observed; vinegar application appeared to reduce venom-induced hemolysis when applied onto adherent tentacles, although the reduction was not significant (Figure 6 and Table 4). Our array allowed us to evaluate vinegar in several contexts, thus solidly evaluating its actual efficacy. Thus, our findings support the use of vinegar as a primary first aid approach for box jellyfish envenomations.

In addition to vinegar, several other first aid approaches showed merit. Pre-treatment and post-treatment with Sting No More™ significantly reduced venom-induced hemolysis (Figure 8 and Table 5). Similarly, Sting No More™ spray was the most effective rinse solution (Figure 6). The principal ingredient in Sting No More™, copper gluconate, prevented cnidae discharge and reduced hemolysis when applied on the tentacle directly (Figure 5 and Figure 6), yet our models revealed that other ingredients in the cream and spray are necessary for efficient delivery across a skin-like barrier (Figure 5 and Figure 8). Lidocaine, a preferred option by some because of its analgesic properties, was effective at inhibiting cnidae discharge (Figure 5 and Table 4), however, it failed to prevent chemically-induced or pressure-induced cnidae discharge (Table 3) and did not significantly reduce hemolysis as a rinse solution (Figure 6 and Table 4) or inactivate venom compounds when applied as a post-sting treatment (Figure 8 and Table 4). We would caution that by masking pain, lidocaine may be harmful; the victim and their medical evaluators may not realize the severity of a sting is if a reduction in pain at the site of the wound gives the false impression of a mild envenomation.

**Table 4:** Summary of research on first-aid treatments and results from this study ± Standard Error.

First Aid	Summary of Published Cnidarian Sting Literature				Formulation in this Study	TSA (% Discharge)	TSBAA (Lysis % Area)	
	Nematocyst Discharge		Pain				Rinse Solution	Post-Sting Treatment
Freshwater	C	+ [28]	C	0 [28], 0 [33]	Double distilled water (ddH_2_O)	C 29.6 ± 7.6	C 34.9 ± 2.7 ^†^	C 23.5 ± 3.0
	S	+ [28]	S	0 [28], 0 [33]				
	H	0 [36], 0 [40]	H	N.D.				
Seawater	C	0 [28], 0 [29], 0 [30], 0 [31]	C	– [28], 0 [33]	Autoclaved Instant Ocean	C 1.8 ± 1.8	C 22.8 ± 1.6	C 29.0 ± 4.5
	S	0 [28], 0 [33], 0 [34]	S	– [28], 0 [33]				
	H	0 [33], 0 [39]	H	0 [38]				
Vinegar (Weak Acetic Acid)	C	– [28] (no test for inhibition), – [29], – [30], – [31], – [32]	C	0 [28], – [31], + [33] (5%), 0/– [42]	Distilled white vinegar	C 0.3 ± 0.3	C 9.0 ± 2.7	C 12.1 ± 2.8
	S	+ [28], 0 [33], – [34], + [35], + [41]	S	+ [28], + [33] (5%)				
	H	+ [33], – [35], + [36], 0/+ [37], –39, 0/+ [40]	H	– [38], – [39]				
Lidocaine	C	0 [28] (Xylocaine®; no test for inhibition)	C	– [28] (see note), – [33]	4% lidocaine-HCl in 110 mM saline	C 0.2 ± 0.2	C 9.2 ± 4.6	C 22.5 ± 6.8
	S	– [33], – [34] (1%)	S	– [28] (see note), – [33]				
	H	– [33]	H	N.D.				
Urine	C	+ [29]	C	N.D.	Human, fresh	C 56.1 ± 15.6 ^δ^	C 17.4 ± 1.4	C 23.3 ± 2.2
	S	0 [33] (10% urea)	S	N.D.				
	H	0 [33] (10% urea), 0/+ [40]	H	N.D.				
Carbonated Cola (Soda)	C	– [31]	C	– [31]	Regular Coca-Cola	C 7.3 ± 1.4	C 23.7 ± 1.9	C 21.9 ± 7.7
	S	N.D.	S	N.D.				
	H	N.D.	H	N.D.				
Ethanol	C	+ [28], + [29] (20%–100%)	C	+ [28], + [33]	70% in ddH_2_O	C 23.2 ± 5.6	C 16.5 ± 1.3	C 28.5 ± 6.8
	S	+ [28], + [33], – [34], – [41]	S	+ [28], + [33]				
	H	+ [33], + [36], +[39], + [40] (Methylated Spirits)	H	+ [38] (Methylated Spirits)				
Epsom salts	N.D.	N.D.	N.D.	N.D.	Saturated in ddH_2_O	C 18.9 ± 7.8	C 13.8 ± 4.1	C 42.9 ± 3.1 ^†^
Isopropanol	C	+ [28]	C	+ [28]	Household (70%)	C 81.2 ± 3.7 ^δ^	C 25.9 ± 4.0	C 30.7 ± 2.5 *
			S	+ [28]				
Heat	N.D.	N.D.	C	– [42], – [43], – [44], – [45]	42 °C–45 °C water pack	not performed	not performed	C 2.1 ± 2.0
			S	N.D.				
			H	N.D.				
Cold	N.D.	N.D.	C	0/– [42], 0 [43], 0 [44], 0 [45]	Ice water pack	not performed	not performed	C 11.9 ± 4.9
			S	N.D.				
			H	– [46]				

Letters indicate class of organism (C for cubozoa, S for schyphozoa, and H for hydrozoa). A “+” indicates increase in nematocyst discharge or worsening of pain; “0” indicates no effect; and “–” indicates prevention of nematocyst discharge or reduction in pain. Superscript numbers indicate references, and percentages in parentheses indicate concentrations used in previous studies, if different from the current study. N.D. indicates not done in any published study to our knowledge. Results presented as percent of cnidae discharged after ten minutes for TSA and hemolytic zone size (percent of total area) for TSBAA. Significant differences were found between treatments in both the TSA and TSBAA (one-way ANOVA *p* < 0.05); Holm-Sidak *post hoc* multiple comparisons indicated as follows: ^δ^ indicates significantly more discharge than seawater in TSA; * indicates significantly lower values than no treatment in TSBAA; **^†^** indicates significantly higher values than no treatment in TSBAA. Note for [28]: limited sample size (2 per condition) and no statistical analyses performed. Also, study used Xylocaine® (lidocaine) dissolved in ethanol, banana essence, methanol, saccharine, macrogol 400 (polyethelene glycol), and purified water (AstraZeneca, North Ryde, NSW, Australia), thus it is impossible to conclude that effects seen are specifically due to lidocaine rather than other potentially inhibitory components such as polyethelene glycol.

**Table 5:** Summary of commercially available first-aid treatments used in this study, including active ingredients and claims, and results from TSBAA ± Standard Error (C for cubozoa).

Name	Manufacturer	Active Ingredients	Claims	Lab or Clinical Investigations	TSBAA (Lysis % Area ± SEM)
Jellyfish Squish^®^	Coastal Solutions, Inc	4% Lidocaine HCl	Prevents skin lesions; much or complete relief of pain within minutes [49]	Studies mentioned on website (no peer-reviewed references)	Post-Sting Treatment: C 14.0 ± 4.7
StingMate^®^	First Aid Mates, LLC	5% acetic acid, menthol	Pain relief within minutes; reduction in skin pathology [50]	Studies mentioned on website (no peer-reviewed references)	Post-Sting Treatment: C 32.9 ± 2.6 ^†^
Safe Sea^®^	Nidaria Technology Ltd	Octinoxate 7.5%, Octixalate 5%, Zinc Oxide 5%, Titanium Dioxide 2%	Prevents the stimulation of jellyfish tentacles after contact with human skin [51]	Relative risk reduction of 82% when compared with normal sunscreen (only 13 stings total) ([52,53,54], See Note A)	Preventative: C 27.9 ± 6.3
Sting No More™ (spray)	Alatalab Solutions, LLC	30 mM copper gluconate, 350 mM urea, 30 mM magnesium sulfate, 3% acetic acid	Relieves jellyfish stings and fire ant bites [55]	This study	Rinse Solution: C 2.5 ± 0.2 *
					Post-Sting Treatment: C 3.9 ± 1.7 *
Sting No More™ (cream)	Alatalab Solutions, LLC	50 mM copper gluconate, 180 mM magnesium sulfate, 10% urea, in a pharmaceutical-grade, non-greasy dermal base	Relieves jellyfish stings and fire ant bites [55]	This study	Preventative: C 1.4 ± 0.3 *

Note A: Prevention of pain and erythema from *Chrysaora fuscescens* but only partly reduced symptoms when *Chiropsalmus quadrumanus* was used; ineffective in an additional small trial [52,53,54]; * indicates significantly lower values than no treatment; ^†^ indicates significantly higher values than no treatment.

While our approach aims to be the most comprehensive and rigorous evaluation of first-aid measures to date, important limitations remain. Specifically, *ex vivo* assay of cytolytic activity may not capture all pathophysiological outcomes of envenomation, in that dermal inflammation due to the trauma of impalement by thousands of foreign nematocyst tubules is likely unaffected by these first-aid treatment solutions and approaches. Further, other venom constituents may not be assessed by these techniques. That said, other studies clearly indicate that the fastest-acting constituent in cubozoan venoms are the cytolytic porins, and that these are necessary and sufficient to recapitulate lethal outcomes.

## 3. Experimental Section

In an effort to develop methodologies that can be employed broadly in other laboratories with other cnidarian species, a tiered research approach was designed to examine a broad array of commonly used first-aid measures, as well as commercial preparations (see Table 1 and Table 2). Instead of forcing nematocysts to fire, our novel models capitalize on the spontaneous stinging response of live tentacles in the presence of blood cells. Venom activity was measured by hemolysis of live blood cells, thus using a well-documented primary venom activity, while more accurately recreating the natural stinging action that occurs during an envenomation event.

The solutions used in all assays are as follows: seawater (Instant Ocean formulated at 35 ppt; Spectrum Brands Inc., Backsburg, VA, USA), freshwater (double-distilled), vinegar (white distilled; Bakers and Chefs CJ314, SAM’s West Inc., Bentonville, AR, USA), lidocaine (4% in 150 mM saline; MP Biomedicals LLC, Solon, OH, USA ), ethanol (Pharmco-Aaper, Brookfield, CT, USA), isopropanol (Fisher Scientific, Fair Lawn, NJ, USA), Epsom salts (CVS Pharmacy Inc., Woonsocket, RI, USA, used saturated in double-distilled water), copper gluconate (30 mM in 150 mM saline; Strem Chemicals, Newburyport, MA, USA).

### 3.1. Tentacle Solution Assay (TSA) to Evaluate Nematocyst Discharge

To determine the effects of first-aid treatments on nematocyst discharge, 15 μL of each solution was added to a coverslip-bottomed slide well containing a 5 mm piece of live, freshly cut tentacle (for a complete list of treatments tested, see Table 1). Video and still images were recorded using a dissecting microscope. For pre-treatment experiments, tentacles were allowed to incubate in the solution for 30 min. In a separate set of wells, tentacle pieces were pre-treated with 15 μL of test solutions for 1 min. The test solution was then removed and 15 μL of isopropanol was added to determine if the solutions irreversibly prevented cnidae discharge.

### 3.2. *Ex Vivo* Assays to Evaluate Hemolytic Activity

We utilized live human RBC from normal donors (approved protocol CHS#12561, University of Hawaii Committee on Human Studies) and low melting point agarose to constitute a live red blood cell agarose to measure hemolysis, a well-documented venom activity [19,20,21]. To create the blood agarose, fresh human RBC were washed and resuspended in modified RPMI (“YRPMI”: 23.81 mM NaHCO_3_, Fisher; 102.67 mM NaCl, BDH; 5.37 mM KCl, Fisher; 0.41 mM MgSO_4_•7H_2_O, Fisher; 25 mM HEPES, Fisher; 6.67 mM NaH_2_PO_4_, Fisher; 0.42 mM Ca(NO_3_)_2_•4H_2_O, Fisher) at 3% and kept at 37 °C. Low melting point, molecular grade agarose (Nusieve GTG Agarose, Lonza, Rockland, ME, USA) was dissolved in YRPMI at 60 °C then cooled to 39 °C. Equal volumes were mixed (final concentrations: 1.5% RBC, 1.5% agarose in YRPMI) and the RBC-agarose solution was immediately aliquotted onto glass to form uniform rectangles as desired and placed at 30 °C to gel. Slides were then maintained in a humidified tissue culture incubator at 37 °C within 4% CO_2_.

The same blood agarose was used for the Venom Blood Agarose Assay (VBAA) and Tentacle Blood Agarose Assay (TBAA). Since some test solutions exhibited direct pH and osmotic effects, we also designed a “skin” layered blood agarose model (Tentacle + Skin Model Blood Agarose Assay or “TSBAA” model). This model also initiated spontaneous stinging in live tentacles. Live time microscopic examination of both tentacle model systems revealed that nematocyst tubules forcibly ejected into the blood agarose layer to result in localized clear nascent zones of lysis of live human RBC. The objective was to develop a reproducible model to recapitulate authentic envenomation amendable to direct assessment of “venom load” or dose via quantification of a known venom activity: hemolysis.

#### 3.2.1. Venom Blood Agarose Assay (VBAA)

Wells 2 mm in diameter were made in 2 mm thick agarose using a biopsy punch (Sklar Instruments, West Chester, PA, USA). Five microliters of venom or diluted venom (serial dilution using 0.5 M citrate) was added to each well and incubated at 37 °C. Photos of lytic zones were taken at 1 h and overnight. The volume of the lytic zone was calculated using the three radii in ImageJ (U.S. National Institutes of Health, Bethesda, MD, USA) used to determine the volume of the cylindrical ring of lysis around the well (cylinder volume minus well volume). These data were then used to determine dose response curve seen in Figure 2 using known hemolytic units [19], the linear portion of which was determined for the calculations in Figure 3.

#### 3.2.2. Tentacle Blood Agarose Assay (TBAA)

Approximately 1 cm of freshly cut tentacle was applied to each slide: tentacles were weighed after the experiment. Slides were then incubated at 37 °C. Images were recorded using a high-resolution scanner (Epson Perfection V500 Photo Scanner, Long Beach, CA, USA), dissecting- or inverted- microscope (Olympus model SZX16 or CKX41SF, Olympus Corporation, Tokyo, Japan) at specific time points. The area of the zone of hemolysis was calculated using ImageJ. Briefly, scale was set using the known slide width and 15 mm × 15 mm subsections were taken from each slide for analysis to remove edge effects. Control slides for each solution (solutions applied without tentacles) were used to set the color threshold for no hemolysis. The percent area of the hemolytic zone was taken directly from the “analyze particles” function. Zone of hemolysis was calculated as the percent total area lysed (Figure 4).

#### 3.2.3. Tentacle Skin Blood Agarose Assay (TSBAA)

Because vinegar and other potential agents led to direct osmotic- or pH-based hemolysis and to better recapitulate the tissue layers involved in authentic tentacle envenomation, we designed a modified blood agarose model with a protective skin layer. Blood agarose slides were prepared as described in the TBAA model but overlaid with a “skin” comprised of pig small intestine modeled after previous published methods for preparation of skin grafts [56]. Specifically, porcine small intestine sections were rinsed in 50 mM saline and sectioned and cut along their length to create thin, flat sheets. Sections were sterilized in 5% Hydrogen peroxide and 10% ethanol in 50 mM saline for two hours, then rinsed in sterile YRPMI three times for 30 min each. Intestinal sections were then stretched over glass to create a thin, flat sheet and allowed to air-dry. The resultant membrane was rubbed with pharmaceutical grade anhydrous lanolin and placed atop the blood agarose squares; these could be removed easily.

In the pre-treatment condition, creams were applied to both sides of the skin and allowed to penetrate for ten minutes. Pre-treated skins were then applied to agarose squares, and approximately 1 cm of tentacle was allowed to spontaneously sting for five minutes.

To test efficacy as tentacle removal solutions, fresh tentacles were allowed to sting for three minutes before 50 μL of the test solution was applied directly onto the tentacle. The treated tentacles remained for another two minutes (for a five-minute total sting time) before the skin was removed.

Lastly, to test efficacy as post-sting treatments, tentacles were allowed to sting spontaneously for five minutes, and 50 μL of sting treatment solutions were added to the skin after tentacle removal. The post-treated skins were removed after two minutes.

All slides were placed at 37 °C for one hour, and the area of the zone of hemolysis was calculated using ImageJ as described for the TBAA (Figure 6). Significant differences between treatments were tested using one-way ANOVAs with Holm-Sidak planned multiple comparison tests to examine differences between tested treatments and no treatment [57].

## 4. Conclusions

The recent debate about the efficacy of vinegar, based on the study by Welfare and co-workers [45], and the related press release-extrapolated claim that vinegar application increases venom load and thus “can kill” demonstrates how poor experimental design models which lack appropriate rigorous controls, would fail to meet fundamental validation criteria. By using newly developed *ex vivo* envenomation models, we have demonstrated that vinegar inhibits nematocyst discharge and that copper gluconate-based products (Sting No More™) inhibit hemolytic activity subsequent to stings of the cubozoan *Alatina* *alata* when used before, during, or after a sting event. Common lay approaches, including urine, were not significantly better than seawater. Based on these data, we conclude that current first-aid protocols for jellyfish stings, including the removal of tentacles by the application of vinegar, do prohibit further cnidae discharge and do not elicit adverse outcomes. Lidocaine has been shown in previous studies to slightly lessen pain and to inhibit nematocyst discharge [33]; that was not confirmed in this study. These data also suggest that while vinegar can and should be used as tentacle-removal solution as it prevents both chemically- and pressure-induced cnidae discharge, it should not be considered an effective treatment for the sting itself. Because of the profound cnidae discharge induced by ethanol and isopropanol and their failure to reduce hemolysis when applied to adherent tentacles, these data do not support their use as treatments. The potent inhibitory effects of copper gluconate-based products (Sting No More™) to hemolytic activity subsequent to stings of the cubozoan *Alatina* *alata* stings when used before, during or after a sting event provides a promising new therapeutic tool for the treatment of cnidarian envenomation.
